# Effect of maternal pre-pregnancy BMI and weekly gestational weight gain on the development of infants

**DOI:** 10.1186/s12937-019-0432-8

**Published:** 2019-01-23

**Authors:** Chao Li, Lingxia Zeng, Duolao Wang, Shaonong Dang, Tao Chen, Victoria Watson, Hong Yan

**Affiliations:** 1 Department of Epidemiology and Biostatistics, School of Public Health, Xi’an Jiaotong University Health Science Center, Xi’an, China; 2 Department of Clinical Sciences, Liverpool School of Tropical Medicine, Pembroke Place, Liverpool, UK; 3 Nutrition and Food Safety Engineering Research Center of Shaanxi Province, Xi’an, China; 4 Key Laboratory of Environment and Genes Related to Diseases, Xi’an Jiaotong University, Xi’an, China; 5 School of Public Health, Xi’an Jiaotong University Health Science Center, No. 76 West Yanta Road, PO Box 46, Xi’an, Xi’an, 710061 Shaanxi China

**Keywords:** Maternal pre-pregnancy BMI, Weekly gestational weight gain, Development of infants

## Abstract

**Objective:**

The aim of the present study is to identify the average effect across different time points and to specify the time effects of maternal pre-pregnancy BMI and weekly gestational weight gain on the mental development and physical growth of infants.

**Methods:**

The present cohort study used a repeated measures study design that began in 2004 with follow up at 3, 6, 12, 18, and 24 months of age. The participants were a subset from a controlled, cluster-randomized, double-blind trial. Bayley Scales of Infant Development (BSID) were used to estimate the mental development of infants. A generalized estimating equation linear model was used to estimate the effects of maternal BMI and weight gain.

**Results:**

The average effect of maternal BMI and weight gain on the weight for age Z scores (WAZ), length for age Z scores (LAZ) and mental development index (MDI) across the different time points of infants was significant. In addition, the maternal BMI and weight gain were positively and significantly associated with the WAZ and LAZ in infants of different ages. However, the effect of weekly gestational weight gain was significant only during the earlier period of life (3 months, Coefficient: 11.15, 95%CI: 4.89–17.41).

**Conclusions:**

Our results indicate positive effects of pre-pregnancy and prenatal nutrition on the physical growth of infants. Weekly gestational weight gain of the pregnant women had a positive effect on the mental development of the infants, but this effect appears to decline over time.

**Electronic supplementary material:**

The online version of this article (10.1186/s12937-019-0432-8) contains supplementary material, which is available to authorized users.

## Introduction

The intellectual development and physical growth in the early life are important for future health and wellbeing. Many prospective studies indicated that mental development was significantly associated with further school achievement, motor and cognitive performance and leadership success and was inversely associated with several health outcomes ascertained in later life [1–4]. Evidence from a series of population-based cohort studies indicated that physical growth in infancy affects the further total and abdominal fat distribution in school-aged children as well as the proximal femoral geometry in adulthood [5, 6].

Recently, the effects of maternal BMI and weight gain on child development have been well studied in higher resource settings. For example, evidence from a cohort study conducted in the United States showed a significant association between the maternal pre-pregnancy BMI and body size of infants at 6 months of age [7]. Another US cohort study found a positive effect of maternal weight gain during pregnancy on the prevalence of overweight children 3 years of age [8]. A large prospective, population-based birth cohort study conducted in the United Kingdom reported a significant association between the maternal pre-pregnancy BMI and children’s cognitive performance [9]. Another UK cohort study reported that the gestational weight gain was positively associated with the school entry assessment scores of children at 4 years of age [10]. However, there is a lack of robust data on the effects of maternal BMI and weight gain in a lower resource setting where there are concerns that inadequate in utero nutrition may have adverse effects on children’s development. In addition, the comprehensive evaluation of the average effect on the mental development and physical growth of infants has rarely been studied.

Therefore, the present cohort study conducted in Changwu and Bing County, which used a repeated measures study design, provides prospective data to identify the average effect across different time points and to specify the time effect of maternal pre-pregnancy BMI and weekly gestational weight gain on the mental development and physical growth of infants (3–24 months).

## Methods

### Study design and population

The present study was a subset of a larger cluster-randomized, double blind trial conducted between 2002 and 2006 in the counties of Changwu and Bing in Shaanxi Province, China. Singleton neonates born only in 2004 were included in the present study. The details of this large trial have been described elsewhere [11–13]. The primary aim of this large trial was to compare the differences in birth outcomes (birthweight and neonatal mortality) among the prenatal micronutrient supplementation groups. The prenatal micronutrient supplementation groups were multi-micronutrient (15 minerals or vitamins), folic acid plus iron, and folic acid (control group). The pregnant women from the same village received the same treatment (prenatal micronutrient supplementation) from enrolment until delivery. The RCT was conducted in Changwu and Bing counties, and there were 14 townships and 234 villages in Changwu County and 20 townships and 327 villages in Bing County included in the study. The randomization of villages was stratified by county with a fixed ratio of treatment (1:1:1) and blocking of 15 to ensure geographical balance with an approximately equal distribution of treatments per township.

The baseline information (sociodemographic, menstrual, reproductive, and medical status, and family history) was collected in the large trial. In addition, the pregnant women enrolled in the trial received three free prenatal care checks at different stages of pregnancy, at which the physical examination including blood pressure and weight measurement was undertaken by trained maternal and child health staff. There were 4604 single live births in the trial, and 1305 of the births occurred in 2004.

Due to limited funding, a subgroup of singleton neonates (born in 2004) was included in the present cohort study and were followed up at 3, 6, 12, 18 and 24 months of age. The baseline characteristics among the participants delivered in 2004 and others were compared, and there were no differences in the baseline characteristics (data not shown). A face-to-face interview was conducted with the primary carers to collect information about the morbidity of infants, time spent outdoors per day and feeding practices. The length of the study was specifically designed with the primary goal of detecting moderate differences in the mean mental development index (MDI) among the three prenatal micronutrient supplementation groups. However, this sample was considered sufficient for detecting 5 IQ point differences, which were considered clinically significant between the prenatal micronutrient supplementation groups [14, 15].

### Setting

The present study was conducted in Changwu and Bing counties, which are both situated in the western part of the Shaanxi Province, and the two counties’ total area is approximately 1752 km^2^. In 2007, the total population of the two rural counties was approximately 497,000. The agricultural annual per capita net income was only 1249 yuan and 780 yuan in Bing and Changwu counties, respectively. The two counties were also classified as Type 4 (poorest) rural counties.

#### Measurement

ᅟ

#### Outcomes

During all visits, the anthropometric measurements and mental development (MD) tests were conducted. The weight and length of the infants were measured using standardized methods. Weight was measured to the nearest 10 g on an electronic scale, excluding the weight of infants’ clothes. The scales were checked by calibration with a standard weight (10 kg) at regular 1 mm intervals on the length board. The length for age Z scores (LAZ), weight for age Z scores (WAZ) and weight for length Z scores (WLZ) of infants were calculated using the 2006 WHO reference values [16].

For the MD test, we used the Bayley Scales of Infant Development (BSID), which can be applied to infants between one and 42 months to assess the mental development of this subgroup of infants [17]. The BSID is a global measure of developmental status in infancy according to assessments of the timely attainment of relatively crude infant milestones [18]. The complete BSID consists of 178 mental scale items regarding sensory acuity, discrimination, memory, and early verbal communication [17, 19]. In the present study, the BSID, which was translated into Chinese, was standardized to be culturally appropriate. The reliability and validity of these measures have been evaluated and shown to be satisfactory [13, 20]. The BSID is a standardized test, and a specific start and stop item were set depending on the age and developmental level of the infant [21]. The MD test was administered in the local hospital or the infant’s own home when the infant was not sleepy, restless, or hungry. The examiners had been trained rigorously and demonstrated high levels of consistency with one another [13]. The raw MD scores were scored by the items that the infant passed on the MD scales of the BSID. The raw MD scores could be converted into an MDI by using standard procedures that are based on the norms of Chinese urban children.

#### Exposures

**Fig. 1:**
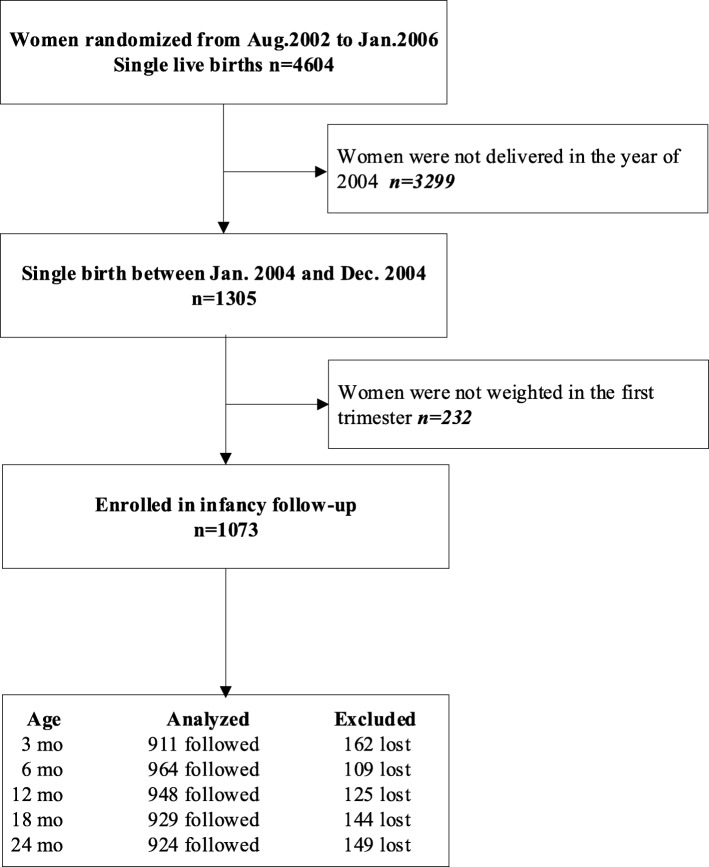
Participant flow chart

In the original large trial, recruited pregnant women could receive three prenatal health checks for free, at which they were asked about complications during pregnancy and were also weighted at the clinic by trained maternal and child health (MCH) staff. The results from a Caucasian study indicated that maternal weight did not change in the first trimester, and changes in maternal weight during pregnancy usually occur after the first trimester [22]. Therefore, maternal BMI during the first trimester is being used as a proxy for pre-pregnancy BMI in the present study. Pre-pregnancy BMI was categorized as underweight (BMI < 18.5), normal-weight (18.5 ≤ BMI < 25), overweight (25 ≤ BMI < 30), or obese (BMI ≥ 30) [23]. The total gestational weight gain was calculated from the maximum weight measured in the third trimester (the last prenatal health check) and the maternal weight was measured in the first trimester (0–14 weeks of gestational age). The gestational age used to calculate the weekly gestational weight gain was calculated from the weeks of gestation in the last prenatal health check in the third trimester minus the weeks of gestation in the first trimester. The weekly gestational weight gain was calculated from the total gestational weight gain divided by the gestational age. The gestational age at birth was measured as completed days based on the first day of the last menstrual period. In the present study, we excluded the participants who were not weighed in the first trimester. Of the 1305 women, 1073 women were weighted in the first trimester, and 1184 women were weighted in the third trimester (Fig. 1).

#### Statistical analysis

All the data were double-entered for verification and checked manually for completeness. Range, extremum, and logical checks were also conducted for accuracy. Statistical significance was set at a *P* value < 0.05 for all statistical tests, and testing was two-sided. The baseline information in the different pre-pregnancy nutritional groups was described by means, standard deviations or percentages. The baseline characteristics of the children and households were compared across the maternal pre-pregnancy nutritional status groups by using ANOVA, chi-square test or fisher exact test. In addition, a wealth index was generated from 16 different household assets or facilities with a principal component analysis method, and this index in the present study was divided into thirds as indicators for the richest, middle income, and poorest households. Due to the repeated measures structure of the data and the original randomized design, the generalized estimating equation (GEE) linear model was used to estimate the effect of maternal pre-pregnancy BMI and weekly gestational weight gain on the physical growth and mental development during the 3–24 months of infant growth. Meanwhile, an interaction between the maternal pre-pregnancy BMI, weekly gestational weight gain and time points of the repeated measurements (months) was added to the models to evaluate the subsequent results at the different time points of the repeated measurements. Estimates of the coefficients and 95% confidence intervals (CIs) were made.

Previous studies found that social factors and prenatal multi-micronutrient supplementation detrimentally affected the physical growth and mental development of children [24–28]. In addition, previous studies reported that higher weight before pregnancy results in less weight gain during pregnancy [29]. Therefore, separate models were fitted to study the effects of maternal weight and weight gain on child development: the model for maternal BMI was not adjusted for maternal weight gain as gestational weight gain is a downstream consequence of maternal weight status, while the model for weekly gestational weight gain was adjusted for maternal pre-pregnancy BMI.

Two models were fitted with multivariable analysis when estimating the effect of maternal pre-pregnancy BMI, with one model adding socio-economic variables (county, sex of infant, maternal age, educational level of parents, household wealth index and occupation of parents) and the other model including the variables of the gestational weeks at birth, gestational weeks when maternal weight was measured, type of prenatal micronutrient supplementation and doses of supplements consumed. When estimating the effect of weekly gestational weight gain, we added one more variable (maternal pre-pregnancy BMI) into the models. In addition, we added another interaction term (prenatal treatment groups and weekly gestational weight gain) into the model as a modification of the effect of the relationship between the gestational weight gain and intellectual development.

To reduce bias and improve the efficiency of the analysis, we used multiple imputation, which has become increasingly popular for handling missing data to estimate the effect of maternal pre-pregnancy BMI and weekly gestational weight gain [30, 31]. The differences between the results of the original GEE model and the imputation model were carefully examined. The data were analysed using STATA software, version 14.0 (Stata Corp LP, College Station, Texas, USA).

## Results

The profile of this cohort study is shown in Fig. 1. In the present study, we excluded infants (17.8%) whose mothers were not weighted in their first trimester. After doing so, the participants in and those excluded from the study did not differ in baseline characteristics (Additional file 1: Table S1). In addition, there were 162 (15.1%), 109 (10.2%), 125 (11.6%), 144 (13.4%), and 149 (13.9%) infants who were lost to follow-up monitoring at 3 months, 6 months, 12 months, 18 months and 24 months, respectively.

Table 1 shows the baseline characteristics of the infants and households in the different maternal pre-pregnancy nutritional status groups. The prevalence of maternal overnutrition (overweight and obesity) and underweight were 2.7% (overweight: 26/1073; obesity: 3/1073) and 17.7% (190/1073), respectively. For the characteristics of children, the highest means of the WAZ, LAZ, WLZ and MDI were in the maternal overweight/obesity group. Compared to the maternal underweight group, the WAZ, WLZ and LAZ of the infants were higher in the maternal normal BMI group. Women who were underweight had the highest weekly weight gain during pregnancy (*P* = 0.003). In addition, the household economic status was best in the women who were overweight or obese, but the difference was not significant (*P* = 0.101).

**Table 1:** Baseline characteristics of children and households by maternal pre-pregnancy nutritional status^a^

	Maternal pre-pregnancy BMI			
	Underweight (BMI < 18.5) *n* = 190	Normal (18.5 ≤ BMI < 25) *n* = 854	Overweight/Obesity (BMI ≥ 25) *n* = 29	P
Child characteristics				
Gender				0.143
Boy	109(57.4)	527(61.7)	22(75.9)	
Girl	81(42.6)	327(38.3)	7(24.1)	
At the age of 3 months				
WAZ	0.43 ± 1.03	0.64 ± 1.05	1.11 ± 1.01	0.005
LAZ	−0.51 ± 1.10	−0.42 ± 1.12	0.01 ± 0.99	0.109
WLZ	1.16 ± 1.19	1.38 ± 1.24	1.54 ± 0.96	0.080
MDI	107.53 ± 10.94	107.92 ± 11.24	109.31 ± 14.55	0.764
At the age of 6 months				
WAZ	0.37 ± 1.10	0.69 ± 1.01	1.25 ± 0.96	< 0.001
LAZ	−0.82 ± 1.24	−0.64 ± 1.21	− 0.16 ± 1.27	0.035
WLZ	1.23 ± 1.24	1.50 ± 1.14	1.82 ± 1.41	0.010
MDI	93.02 ± 16.82	93.54 ± 15.42	94.15 ± 17.85	0.907
At the age of 12 months				
WAZ	0.27 ± 0.96	0.42 ± 1.01	1.05 ± 1.10	0.002
LAZ	−0.64 ± 1.18	−0.52 ± 1.09	0.22 ± 1.01	0.002
WLZ	0.76 ± 1.02	0.89 ± 1.08	1.25 ± 1.11	0.095
MDI	103.26 ± 21.92	102.16 ± 21.80	104.71 ± 21.22	0.731
At the age of 18 months				
WAZ	0.17 ± 0.89	0.34 ± 0.90	0.71 ± 0.88	0.011
LAZ	−0.83 ± 1.12	−0.81 ± 1.09	− 0.38 ± 0.78	0.165
WLZ	0.78 ± 0.91	0.96 ± 0.97	1.19 ± 0.97	0.045
MDI	99.90 ± 19.91	99.55 ± 19.89	106.30 ± 19.43	0.235
At the age of 24 months				
WAZ	0.22 ± 0.91	0.24 ± 0.90	0.75 ± 0.79	0.023
LAZ	−0.62 ± 1.20	−0.64 ± 1.02	− 0.38 ± 0.71	0.492
WLZ	0.87 ± 0.95	0.93 ± 0.97	1.43 ± 1.01	0.032
MDI	89.05 ± 20.44	90.44 ± 17.79	93.18 ± 14.37	0.499
Gestational age at birth, wk.	39.92 ± 1.38	39.93 ± 1.51	39.94 ± 1.47	0.996
Women’s characteristics				
Maternal age, y	24.06 ± 4.09	24.83 ± 4.43	25.46 ± 4.96	0.078
Weekly gestational weight gain, kg/wk	0.39 ± 0.25	0.34 ± 0.19	0.27 ± 0.18	0.003
Women’s education				0.018
Primary	41(21.6)	256(30.0)	9(31.0)	
Secondary	119(62.6)	476(55.9)	20(69.0)	
≥ High school	30(15.8)	120(14.1)	0	
Women’s occupation at enrollment				0.180
Farmer	159(83.7)	713(83.9)	28(96.6)	
Others	31(16.3)	137(16.1)	1(3.4)	
Father’s education				0.427
Primary	19(10.1)	90(10.6)	0	
Secondary	122(64.9)	557(65.4)	21(72.4)	
≥ High school	47(25.0)	204(24.0)	8(27.6)	
Father’s occupation at enrollment				0.569
Farmer	140(73.7)	633(74.2)	24(82.8)	
Others	50(26.3)	220(25.8)	5(17.2)	
Type of prenatal micronutrients supplementation				0.447
Folic acid	36(18.9)	215(25.2)	10(34.5)	
Iron/folic acid	82(43.2)	327(38.3)	6(20.7)	
Multimicronutrients	72(37.9)	312(36.5)	13(44.8)	
Household wealth index at enrollment				0.101
Poorest	69(36.3)	302(35.4)	9(31.0)	
Middle	61(32.1)	299(35.0)	7(24.2)	
Richest	60(31.6)	253(29.6)	13(44.8)	

Abbreviation: *LAZ* Length for age Z scores, *MDI* Mental Development, *WAZ* Weight for age Z scores, *WLZ* Weight for length Z scores

^a^Values are n(%) or means ± SDs. Anova, Chi-square or Fisher exact test were used to compare differences among three groups

Table 2 shows the significant association between the maternal pre-pregnancy BMI and WAZ (total, Coefficient: 0.07, 95%CI: 0.04–0.09), LAZ (total, Coefficient: 0.03, 95%CI: 0.01–0.06) and WLZ (total, Coefficient: 0.06, 95%CI: 0.04–0.08) of infants, but the association between the pre-pregnancy BMI and MDI (total, Coefficient: 0.22, 95%CI: -0.11-0.54) was not significant. In the unadjusted analysis, the maternal pre-pregnancy BMI was significantly associated with the infant WAZ and WLZ at the ages of 3, 6, 12, 18 and 24 months. However, the LAZ was significantly associated with the maternal pre-pregnancy BMI at earlier ages of 3, 6 and 12 months. In addition, the pre-pregnancy BMI was associated with the infant WAZ (Coefficient: 11.15, 95%CI: 4.89–17.41), LAZ (Coefficient: 11.15, 95%CI: 4.89–17.41) and WLZ (Coefficient: 11.15, 95%CI: 4.89–17.41). After adjusting for the variables of county, sex of infant, maternal age, educational level of parents, household wealth index, occupation of parents (Model 1), gestational weeks at birth, gestational weeks when maternal weight was measured, maternal pre-pregnancy BMI, type of prenatal micronutrient supplementation and doses of supplements consumed (Model 2), the effect of the maternal pre-pregnancy BMI was still significant in the different ages of the infants. For the mental development of infants, the association between the maternal pre-pregnancy BMI and MDI of the infants was not significant at any age (3, 6, 12, 18, 24 months). The average effect of the maternal pre-pregnancy BMI was significant on the physical growth of infants but not significant on the mental development of infants, and the results were similar among the unadjusted analysis models, model 1 and model 2. In addition, we found no material differences after adding an interaction term (prenatal treatment groups and weekly gestational weight gain) into the GEE model (Additional file 1: Table S2).

**Table 2:** Association between maternal pre-pregnancy BMI and development of infants ^a^

	Maternal pre-pregnancy BMI					
	Unadjusted analysis		Model 1 ^b^		Model 2 ^c^	
	Coef(95%CI)	*P*	Coef(95%CI)	*P*	Coef(95%CI)	*P*
Age of 3 months						
WAZ	0.07(0.04,0.09)	< 0.001	0.07(0.04,0.10)	< 0.001	0.07(0.05,0.10)	< 0.001
LAZ	0.04(0.01,0.07)	0.027	0.04(0.01,0.07)	0.016	0.04(0.01,0.07)	0.013
WLZ	0.05(0.02,0.08)	0.002	0.06(0.02,0.09)	0.001	0.06(0.02,0.09)	0.001
MDI	0.34(−0.19,0.87)	0.204	0.47(− 0.06,1.00)	0.085	0.39(− 0.14,0.92)	0.145
Age of 6 months						
WAZ	0.08(0.06,0.11)	< 0.001	0.09(0.06,0.12)	< 0.001	0.09(0.06,0.12)	< 0.001
LAZ	0.04(0.01,0.07)	0.015	0.05(0.01,0.08)	0.005	0.05(0.02,0.08)	0.004
WLZ	0.08(0.05,0.11)	< 0.001	0.08(0.05,0.11)	< 0.001	0.08(0.05,0.11)	< 0.001
MDI	−0.07(−0.59,0.44)	0.785	0.13(−0.38,0.65)	0.612	0.07(−0.45,0.58)	0.796
Age of 12 months						
WAZ	0.05(0.02,0.08)	0.001	0.06(0.03,0.08)	< 0.001	0.06(0.03,0.08)	< 0.001
LAZ	0.04(0.01,0.07)	0.021	0.04(0.01,0.07)	0.012	0.04(0.01,0.08)	0.009
WLZ	0.04(0.01,0.07)	0.008	0.04(0.01,0.08)	0.007	0.04(0.01,0.08)	0.006
MDI	0.12(− 0.39,0.64)	0.638	0.35(− 0.17,0.87)	0.188	0.28(− 0.24,0.80)	0.297
Age of 18 months						
WAZ	0.06(0.03,0.09)	< 0.001	0.06(0.03,0.09)	< 0.001	0.06(0.04,0.09)	< 0.001
LAZ	0.01(− 0.03,0.04)	0.694	0.01(− 0.02,0.04)	0.565	0.01(− 0.02,0.04)	0.509
WLZ	0.08(0.04,0.11)	< 0.001	0.08(0.04,0.11)	< 0.001	0.08(0.04,0.11)	< 0.001
MDI	0.01(−0.51,0.53)	0.965	0.13(−0.39,0.65)	0.625	0.06(−0.46,0.58)	0.824
Age of 24 months						
WAZ	0.04(0.01,0.07)	0.005	0.05(0.02,0.08)	0.001	0.05(0.02,0.08)	0.001
LAZ	0.01(− 0.02,0.04)	0.530	0.01(− 0.02,0.05)	0.386	0.02(−0.02,0.05)	0.342
WLZ	0.05(0.02,0.08)	0.002	0.05(0.02,0.08)	0.002	0.05(0.02,0.08)	0.002
MDI	0.13(−0.39,0.66)	0.620	0.35(−0.17,0.88)	0.189	0.39(−0.24,0.81)	0.281
Total						
WAZ	0.06(0.04,0.08)	< 0.001	0.07(0.04,0.09)	< 0.001	0.07(0.04,0.09)	< 0.001
LAZ	0.03(0.01,0.05)	0.044	0.03(0.01,0.06)	0.021	0.03(0.01,0.06)	0.016
WLZ	0.06(0.04,0.08)	< 0.001	0.06(0.04,0.08)	< 0.001	0.06(0.04,0.08)	< 0.001
MDI	0.10(−0.23,0.44)	0.540	0.28(− 0.04,0.61)	0.089	0.22(− 0.11,0.54)	0.196

Abbreviation: *CI* confidence interval, *LAZ* length for age Z scores, *MDI* mental development index, *WAZ* weight for age Z scores, *WLZ* weight for length Z scores

^a^Generalized estimated equation linear model were used to assess the association between maternal pre-pregnancy BMI and development of infants

^b^Model 1 include the variables of county, sex of infant, maternal age, educational level of parents, household wealth index, occupation of parents

^c^Model 2 include the variables of county, sex of infant, maternal age, educational level of parents, household wealth index, occupation of parents, gestational weeks at birth, gestational weeks when maternal weight measured, maternal pre-pregnancy BMI, type of prenatal micronutrient supplementation, doses of supplements consumed

A similar analysis was conducted to estimate the relationships between the weekly gestational weight gain and physical growth (WAZ, LAZ and WLZ) and the mental development (MDI) of infants. Overall, there was a significant association between the weekly gestational weight gain and WAZ (Coefficient: 0.38, 95%CI: 0.09–0.66) as well as the LAZ (Coefficient: 0.14, 95%CI: 0.12–0.76) and MDI (Coefficient: 5.01, 95%CI: 1.03–8.99) of infants, but the association between the weekly gestational weight gain and WLZ (Coefficient: 0.14, 95%CI: -0.12-0.41) was not significant. For physical growth, the results from models 1 and 2 showed a significant association between the weekly gestational weight gain and physical growth (WAZ and LAZ) at different time points of measurement. For mental development, the effect of weekly gestational weight gain was significant in the earlier period of life (3 months, Coefficient: 11.15, 95%CI: 4.89–17.41, Model 2). The average effect of weekly gestational weight gain across the different time points was significant in the WAZ (Coefficient: 0.38, 95%CI: 0.09–0.66, Model 2) and LAZ (Coefficient: 0.44, 95%CI: 0.12–0.76, Model 2) after controlling for the confounders. In addition, the average effects of weekly gestational weight gain were significant for the mental development (Coefficient: 5.01, 95%CI: 1.03–8.99, Model 2) of infants. The results were similar among the unadjusted and multivariable analyses (Table 3). A sensitivity analysis was conducted using multiple imputation methods, and there were no material differences between the results of the sensitivity and original analyses (Additional file 1: Table S3, Additional file 1: Table S4).

**Table 3:** Association between gestational weekly weight gain and development of infants ^a^

	Weekly gestational weight gain					
	Unadjusted analysis		Model 1 ^b^		Model 2 ^c^	
	Coef(95%CI)	*P*	Coef(95%CI)	*P*	Coef(95%CI)	*P*
Age of 3 months						
WAZ	0.38(0.06,0.71)	0.022	0.37(0.04,0.70)	0.027	0.37(0.03,0.70)	0.034
LAZ	0.42(0.04,0.80)	0.029	0.40(0.02,0.78)	0.039	0.42(0.03,0.81)	0.036
WLZ	0.01(−0.37,0.39)	0.946	0.02(−0.36,0.39)	0.936	0.03(−0.35,0.41)	0.862
MDI	10.49(4.82,17.03)	< 0.001	10.81(4.75,16.88)	< 0.001	11.15(4.89,17.41)	< 0.001
Age of 6 months						
WAZ	0.38(0.06,0.71)	0.022	0.38(0.05,0.71)	0.024	0.37(0.04,0.71)	0.031
LAZ	0.59(0.21,0.97)	0.002	0.57(0.19,0.95)	0.004	0.59(0.19,0.98)	0.003
WLZ	−0.05(−0.44,0.33)	0.779	− 0.04(− 0.42,0.33)	0.819	− 0.03(− 0.41,0.36)	0.895
MDI	4.54(− 1.52,10.60)	0.142	4.85(− 1.17,10.88)	0.114	4.57(− 1.63,10.78)	0.148
Age of 12 months						
WAZ	0.52(0.19,0.84)	0.002	0.51(0.18,0.84)	0.002	0.51(0.17,0.84)	0.003
LAZ	0.41(0.03,0.79)	0.034	0.40(0.02,0.78)	0.038	0.40(0.01,0.80)	0.043
WLZ	0.37(−0.01,0.75)	0.054	0.38(0.01,0.75)	0.045	0.40(0.02,0.78)	0.039
MDI	5.04(−1.01,11.09)	0.103	5.07(−0.94,11.08)	0.099	5.85(−0.36,12.05)	0.065
Age of 18 months						
WAZ	0.30(−0.03,0.63)	0.077	0.29(−0.04,0.63)	0.088	0.28(−0.06,0.63)	0.105
LAZ	0.43(0.05,0.82)	0.028	0.40(0.02,0.79)	0.041	0.42(0.02,0.81)	0.040
WLZ	0.09(−0.30,0.48)	0.651	0.10(−0.29,0.48)	0.618	0.12(−0.27,0.50)	0.561
MDI	4.08(−2.05,10.21)	0.192	4.27(−1.83,10.37)	0.170	4.36(− 1.94,10.66)	0.175
Age of 24 months						
WAZ	0.36(0.03,0.69)	0.032	0.35(0.02,0.68)	0.040	0.34(0.01,0.68)	0.049
LAZ	0.40(0.01,0.78)	0.043	0.37(−0.02,0.75)	0.062	0.38(− 0.02,0.77)	0.061
WLZ	0.16(−0.22,0.55)	0.411	0.17(−0.21,0.55)	0.380	0.19(−0.20,0.58)	0.336
MDI	−0.85(−6.96,5.27)	0.785	−1.08(−7.17,5.00)	0.727	−0.65(−6.93,5.63)	0.839
Total						
WAZ	0.39(0.12,0.66)	0.005	0.38(0.11,0.65)	0.006	0.38(0.09,0.66)	0.009
LAZ	0.45(0.15,0.76)	0.004	0.43(0.13,0.73)	0.006	0.44(0.12,0.76)	0.006
WLZ	0.12(−0.15,0.38)	0.381	0.12(− 0.13,0.38)	0.333	0.14(− 0.12,0.41)	0.284
MDI	4.70(0.79,8.60)	0.018	4.74(0.93,8.55)	0.015	5.01(1.03,8.99)	0.014

Abbreviation: *CI* Confidence interval, *LAZ* Length for age Z scores, *MDI* Mental development index, *WAZ* Weight for age Z scores

^a^Generalized estimated equation linear model were used to assess the association between weekly gestational weight gain and development of infants

^b^Model 1 include the variables of county, sex of infant, maternal age, educational level of parents, household wealth index, occupation of parents

^c^Model 2 include the variables of county, sex of infant, maternal age, educational level of parents, household wealth index, occupation of parents, gestational weeks at birth, gestational weeks when maternal weight measured, maternal pre-pregnancy BMI, type of prenatal micronutrient supplementation, doses of supplements consumed

## Discussion

In the present study, we found the effects of maternal pre-pregnancy and weekly gestational weight gain on the physical and mental development of infants across different time points. In addition, the maternal pre-pregnancy BMI and weekly gestational weight gain were positively associated with the physical growth (WAZ and LAZ) of infants of different ages. The effect of the weekly gestational weight gain on the offspring’s mental development (MDI) tended to decline as the infant became older.

### Comparison with other studies

Previous studies have reported the effect of maternal pre-pregnancy BMI on child development. For physical growth, evidence from a large cohort study conducted in Norway showed a positive association between the maternal pre-pregnancy BMI and offspring BMI at 3 years of age [32]. Results from an Iranian cross-sectional study showed the positive effects of pre-pregnancy body mass index on birth weight, where a low pre-pregnancy BMI was found to be a risk factor for low birth weight [33]. Another prospective pregnancy cohort study conducted in the United States found that a higher pre-pregnancy BMI was associated with higher WAZ and weight-for-length z scores of infants [7]. For mental development, a prospective study focusing on African-American children found that pre-pregnancy obese women were at risk of having children (mean age of 5.3 years) with diminished intellectual ability (general IQ and nonverbal scores) [34]. Evidence from another cohort of children born in Finland showed that pre-pregnancy maternal obesity (BMI ≥ 30) was a risk factor for intellectual disability in adults aged 20 years, but a similar association was not found among children at the age of 11.5 years [35]. A large prospective cohort study in the United Kingdom found a negative effect of maternal pre-pregnancy BMI on children’s cognitive performance at 5 and 7 years of age [9]. In the present study, the average effect of maternal pre-pregnancy BMI on the physical growth (WAZ and LAZ) of infants across different time points was identified. This result was consistent with the findings of other previous studies. An effect of maternal pre-pregnancy BMI on the mental development of infants was not found in the present study. However, the present study was conducted in poor areas of China where the incidence of maternal overweight or obesity is only 2.7%, but the incidence of maternal underweight is 17.7%. Conversely, the incidence of maternal overnutrition (overweight or obesity) and underweight in the UK are 29 and 5.4%, respectively [9]. Therefore, the differences in the incidences of the maternal mothers who are underweight and over-nourished between poor areas in China and the UK may explain the non-significant effects of maternal pre-pregnancy BMI observed in the present study. Our present findings emphasize the importance of maternal pre-pregnancy nutritional status on the prevention of infants’ undernutrition, especially in poor areas.

Many studies have also reported the importance of maternal weight gain during pregnancy on children’s development. Specifically, many studies found an adverse effect of excessive or lower weight gain during pregnancy. Evidence from a cross-sectional study in Portugal showed that excessive gestational weight gain was significantly associated with a higher risk of overweight offspring in young school-aged children [36]. However, the recall bias of the Portuguese study could not be avoided due to the cross-sectional study design. A longitudinal study conducted in the United States reported that excessive gestational weight gain was associated with an increase in the offspring BMI z-scores at 5 years of age [37]. Another US cohort study reported a similar association between the excessive gestational weight gain and the risk of children aged 7 years who were overweight [38]. Findings from a prospective study showed that inadequate gestational weight gain was significantly associated with increased odds of being small-for-gestational-age and decreased odds of postpartum weight retention. Excessive gestational weight gain was associated with a decreased risk of being small-for-gestational-age, increased odds of postpartum weight retention, and overweight children [39]. For mental development, a large prospective, population-based birth cohort study conducted in the United Kingdom reported that gestational weight gain was positively associated with IQ (at 8 years of age) and school entry assessment scores (at 4 years of age) [10]. Another birth cohort study in Poland reported that excessive gestational weight gain had a negative impact on the cognitive development of children at 7 years of age [40]. The results from the present study were similar to previous studies, as we found a positive and significant average effect of gestational weight gain on the physical growth and mental development of infants, and the effect of gestational weight gain on the physical growth was still significant at different time points of measurement. For the mental development of infants, the effect of maternal weekly gestational weight gain was only significant for the earlier period (3 months) of infants. In addition, because gestational weight gain was used as an indicator of nutritional status during pregnancy [41], an effect of the maternal pre-pregnancy weight was not found on the infants’ mental development. Our results suggest the importance of improving prenatal nutritional status in the further development of children. Most previous studies categorized women as having gained excess, adequate or inadequate weight according to the Institute of Medicine guidelines [41], but the present study could not provide similar results as the times that maternal weight was measured in the first and third trimesters were not fixed.

To the best of our knowledge, most similar previous studies specified the effect of maternal BMI and weight gain on child development at certain ages, and the effect on child development at a specific period of time has rarely been reported, especially in poor areas. A repeated measures study design was used in the present study. Therefore, the average effect and time effect of maternal pre-pregnancy BMI and gestational weight gain could be comprehensively estimated. The present study found a positive effect of maternal pre-pregnancy BMI and gestational weight gain on the physical growth of infants. A possible explanation is that environmental factors, such as pre-pregnancy and prenatal lifestyle, including physical activity and food intake, which affect maternal BMI and gestational weight gain, were also reported to contribute to offspring growth [42]. These findings are consistent with activity interventions during pregnancy. For the mental development of infants, a significant association between the maternal pre-pregnancy BMI and MDI was not found at any age of the infants. A possible explanation for this could be the weak effect of pre-pregnancy nutritional status on the mental development of infants. Our sample was not large enough to detect such a small association between the maternal BMI and MDI. Our results showed that the effect of gestational weight gain on the MDI was only significant in infants at 3 months. In addition, the infants in the present study were followed until they reached an early school age, and a significant association between the gestational weight gain and mental development in the early school-aged children was not found (data not shown). Our previously published results showed the positive effect of prenatal multi-micronutrient supplementation on the mental development of infants at 1 year of age, but the association between the prenatal micronutrient supplementation and the mental development of early school-aged children was found to be nonsignificant. These results support the idea that the positive effects of prenatal nutrition on child mental development may fade over time. A possible explanation for this could be the present study was conducted in the poor areas in China. Evidence suggests that lower level of early educational exposure and nutritional advantages are available to rural infants as opposed to their urban counterparts [43, 44]. These postnatal factors that have been proved associated cognitive development of infants [45] might cover the effect of weekly gestational weight gain. In addition, recent animal studies have reported an association between the prenatal nutrition and mental development. Malnutrition appears to alter cell migration, cell numbers, synaptogenesis, myelinisation, neurotransmission and hippocampal formation in rats [46–49].

### Strengths and limitations

The strengths of the study are its standardized measurement procedures and its prospective data collection. In addition, the repeated measures study design enabled us to comprehensively evaluate the average effect across different time points and the time effect of maternal BMI and gestational weight gain on child development. Our study also has several limitations. First, there were a few missing values in the variables of maternal weight and child development. After excluding the participants who were not weighted in the first trimester, we compared the baseline characteristics between the excluded and included groups. We found that the two groups did not differ in their baseline characteristics (Additional file 1: Table S1). In addition, we used a multiple imputation method as a sensitivity analysis, and we found that the results were robust after comparing the differences between the sensitivity analysis and the original analysis (Additional file 1: Tables S3 and S4). Second, we did not include all possible confounders. For example, we did not collect information of maternal dietary during pregnancy and lactation and parental mental development which is a strong factor of offspring mental development [50] in addition to maternal psychological distress which may be associated with parenting behaviour. However, the results from a large cohort study showed that the significant association between maternal behavioural problems and offspring weight is not affected by the adjustment for maternal depression and anxiety [51]. Despite this, consistent outcomes after the adjustment for different confounders also proved present results to be reliable and robust. Although there were several limitations in the present study, this study contributes to the literature by providing robust data on the effects of maternal weight and weight gain in a poor area where there are concerns that inadequate in utero nutrition may have adverse effects on child development.

## Conclusion

In conclusion, we identified the positive effect of pre-pregnancy BMI and gestational weight gain on the physical growth of infants. For the mental development of infants, the effect of weekly gestational weight gain was identified, but the effect appears to fade away over time. Our findings emphasized the importance of improving pre-pregnancy and prenatal nutrition on infants’ development in a lower resource setting.

## Additional file

Additional file 1: **Table S1.** Baseline characteristics of participants in excluded and included groups. **Table S2.** Association between gestational weekly weight gain and development of infants. **Table S3.** Association between maternal pre-pregnancy BMI and development of infant (sensitivity analysis, multiple imputation method). **Table S4.** Association between gestational weekly weight gain and development of infant (sensitivity analysis, multiple imputation method). (DOCX 33 kb)

## Abbreviations

LAZ: Length for age Z scores
MDI: Mental development index
WAZ: Weight for age Z scores
WLZ: Weight for Length Z scores
