# Supplementary material for: Effect of maternal pre-pregnancy BMI and weekly gestational weight gain on the development of infants
Source: Nutr J. 2019 Jan 23;18:6. doi: 10.1186/s12937-019-0432-8 (PMC6345052; doi:10.1186/s12937-019-0432-8)
Supplement: Supplementary file 1 — Table S1. Baseline characteristics of participants in excluded and included groups. Table S2. Association between gestational weekly weight gain and development of infants. Table S3. Association between maternal pre-pregnancy BMI and development of infant (sensitivity analysis, multiple imputation method). Table S4. Association between gestational weekly weight gain and development of infant (sensitivity analysis, multiple imputation method). (DOCX 33 kb) [file 12937_2019_432_MOESM1_ESM.docx]

| **Table S1**  Baseline characteristics of participants in excluded and included groups ^1^ | | | |
| --- | --- | --- | --- |
|  | Groups | | *P* |
|  | Included | Excluded |  |
| **Child characteristics** |  |  |  |
| Number of children | 1073 | 232 |  |
| Birth weight, kg | 3.19±0.41 | 3.16±0.42 | 0.262 |
| Gender |  |  | 0.690 |
| Boy | 658(61.3) | 139(59.9) |  |
| Girl | 415(38.7) | 93(40.1) |  |
| Gestational age at birth, wk | 39.93±1.48 | 40.11±1.66 | 0.110 |
| **Women’s characteristics** |  |  |  |
| Maternal age, y | 24.17±4.41 | 24.80±4.45 | 0.058 |
| Women's education |  |  | 0.421 |
| <3 years | 44(4.1) | 11(4.8) |  |
| Primary | 262(24.5) | 58(25.1) |  |
| Secondary | 615(57.4) | 139(60.2) |  |
| ≥ High school | 150(14.0) | 23(9.9) |  |
| Women's occupation at enrollment |  |  | 0.212 |
| Farmer | 900(84.2) | 202(87.5) |  |
| Others | 169(15.8) | 29(12.5) |  |
| **Others** |  |  |  |
| Father's education |  |  | 0.155 |
| <3 years | 9(0.8) | 2(0.9) |  |
| Primary | 100(9.4) | 25(10.8) |  |
| Secondary | 700(65.5) | 164(71.0) |  |
| ≥ High school | 259(24.3) | 40(17.3) |  |
| Father's occupation at enrollment |  |  | 0.470 |
| Farmer | 797(74.4) | 177(76.6) |  |
| Others | 275(25.6) | 54(23.4) |  |
| Household wealth index at enrollment |  |  | 0.130 |
| Very poor | 140(13.1) | 24(10.3) |  |
| Poor | 199(18.5) | 45(19.4) |  |
| Middle | 237(22.1) | 63(27.2) |  |
| Rich | 252(23.5) | 61(26.3) |  |
| Very rich | 245(22.8) | 39(16.8) |  |
| ^1^ Values are n(%) or means ± SD | | | |

| **Table S2** Association between gestational weekly weight gain and development of infants ^a^ | | | | | | | | |
| --- | --- | --- | --- | --- | --- | --- | --- | --- |
|  | Weekly gestational weight gain | | | | | | | |
|  | Model 1 ^b^ | |  | Model 2 ^c^ | |  | Model 3 ^d^ | |
|  | Coef(95%CI) | *P* |  | Coef(95%CI) | *P* |  | Coef(95%CI) | *P* |
| **Age of 3 months** |  |  |  |  |  |  |  |  |
| WAZ | 0.36(0.03,0.70) | 0.034 |  | 0.40(0.07,0.73) | 0.018 |  | 0.37(0.04,0.70) | 0.031 |
| LAZ | 0.43(0.05,0.81) | 0.028 |  | 0.41(0.03,0.79) | 0.034 |  | 0.42(0.03,0.81) | 0.035 |
| WLZ | 0.01(-0.36,0.38) | 0.960 |  | 0.05(-0.32,0.42) | 0.775 |  | 0.06(-0.31,0.44) | 0.742 |
| MDI | 10.33(4.22,16.45) | 0.001 |  | 10.07(3.98,16.15) | 0.001 |  | 10.76(4.50,17.02) | 0.001 |
| **Age of 6 months** |  |  |  |  |  |  |  |  |
| WAZ | 0.36(0.03,0.70) | 0.034 |  | 0.41(0.07,0.74) | 0.016 |  | 0.38(0.04,0.72) | 0.028 |
| LAZ | 0.60(0.21,0.98) | 0.002 |  | 0.58(0.20,0.96) | 0.003 |  | 0.59(0.20,0.98) | 0.003 |
| WLZ | -0.07(-0.45,0.30) | 0.691 |  | -0.03(0.40,0.34) | 0.872 |  | -0.02(-0.40,0.36) | 0.915 |
| MDI | 3.99(-2.08,10.06) | 0.198 |  | 4.17(-1.87,10.21) | 0.176 |  | 4.23(-1.97,10.43) | 0.181 |
| **Age of 12 months** | |  |  |  |  |  |  |  |
| WAZ | 0.49(0.16,0.83) | 0.004 |  | 0.54(0.21,0.87) | 0.001 |  | 0.51(0.18,0.85) | 0.003 |
| LAZ | 0.42(0.03,0.80) | 0.033 |  | 0.41(0.03,0.80) | 0.033 |  | 0.41(0.02,0.80) | 0.041 |
| WLZ | 0.33(-0.04,0.70) | 0.081 |  | 0.37(0.01,0.74) | 0.047 |  | 0.38(0.01,0.76) | 0.045 |
| MDI | 4.46(-1.61,10.52) | 0.150 |  | 4.34(-1.68,10.37) | 0.158 |  | 5.50(-0.70,11.70) | 0.082 |
| **Age of 18 months** | |  |  |  |  |  |  |  |
| WAZ | 0.28(-0.06,0.62) | 0.106 |  | 0.32(-0.02,0.65) | 0.065 |  | 0.29(-0.05,0.63) | 0.096 |
| LAZ | 0.44(0.05,0.83) | 0.027 |  | 0.41(0.03,0.80) | 0.037 |  | 0.42(0.02,0.82) | 0.039 |
| WLZ | 0.02(-0.35,0.40) | 0.902 |  | 0.07(-0.31,0.45) | 0.727 |  | 0.08(-0.31,0.46) | 0.694 |
| MDI | 3.47(-2.68,9.61) | 0.269 |  | 3.49(-2.63,9.61) | 0.263 |  | 3.93(-2.37,10.23) | 0.221 |
| **Age of 24 months** | |  |  |  |  |  |  |  |
| WAZ | 0.34(0.01,0.68) | 0.047 |  | 0.37(0.04,0.71) | 0.027 |  | 0.35(0.01,0.69) | 0.044 |
| LAZ | 0.40(0.02,0.79) | 0.042 |  | 0.38(-0.01,0.76) | 0.055 |  | 0.38(-0.02,0.77) | 0.060 |
| WLZ | 0.16(-0.22,0.53) | 0.415 |  | 0.20(-0.18,0.58) | 0.295 |  | 0.21(-0.17,0.59) | 0.277 |
| MDI | -1.51(-7.65,4.63) | 0.630 |  | -1.92(-8.03,4.19) | 0.538 |  | -1.14(-7.42,5.15) | 0.723 |
| **Total** |  |  |  |  |  |  |  |  |
| WAZ | 0.37(0.09,0.65) | 0.010 |  | 0.41(0.14,0.68) | 0.003 |  | 0.38(0.10,0.67) | 0.008 |
| LAZ | 0.46(0.15,0.76) | 0.004 |  | 0.44(0.13,0.75) | 0.005 |  | 0.44(0.13,0.76) | 0.006 |
| WLZ | 0.09(-0.16,0.34) | 0.487 |  | 0.13(-0.12,0.38) | 0.293 |  | 0.14(-0.11,0.40) | 0.274 |
| MDI | 4.10(0.17,8.03) | 0.041 |  | 3.99(0.15,7.83) | 0.042 |  | 4.61(0.64,8.59) | 0.023 |
| Abbreviation: CI, Confidence interval. LAZ, Length for age Z scores; MDI, Mental development index;  WAZ, Weight for age Z scores; WLZ, Weight for length Z scores.  ^a^ Generalized estimated equation linear model were used to assess the association between maternal weight gain  during pregnancy and physical development in early school aged children.  ^b^ Model 1 include two interaction terms (weekly gestational weight gain*treatment groups; weekly gestational  weight gain*age of infants).  ^c^ Model 1 include the variables of county, sex of infant, maternal age, educational level of parents, household  wealth index, occupation of parents and two interaction terms (weekly gestational weight gain*treatment groups;  weekly gestational weight gain*age of infants). | | | | | | | | |
| ^d^ Model 2 include the variables of county, sex of infant, gestational weeks at birth, gestational weeks when  maternal weight measured, type of prenatal micronutrient supplementation, doses of supplements consumed,  maternal age during pregnancy, educational level of parents, household wealth index, occupation of parents  and two interaction terms (weekly gestational weight gain*treatment groups; weekly gestational weight gain*age  of infants). | | | | | | | | |

| **Table S3** Association between maternal pre-pregnancy BMI and development of infant (sensitivity analysis, multiple imputation method) ^a^ | | | | | | | | |
| --- | --- | --- | --- | --- | --- | --- | --- | --- |
|  | Maternal pre-pregnancy BMI | | | | | | | |
|  | Unadjusted analysis | |  | Model 1 ^b^ | |  | Model 2 ^c^ | |
|  | Coef(95%CI) | *P* |  | Coef(95%CI) | *P* |  | Coef(95%CI) | *P* |
| **Age of 3 months** |  |  |  |  |  |  |  |  |
| WAZ | 0.06(0.03,0.09) | <0.001 |  | 0.06(0.03,0.09) | <0.001 |  | 0.06(0.03,0.10) | <0.001 |
| LAZ | 0.03(-0.01,0.07) | 0.058 |  | 0.04(0.01,0.07) | 0.038 |  | 0.04(0.01,0.07) | 0.029 |
| WLZ | 0.04(0.01,0.08) | 0.006 |  | 0.04(0.01,0.08) | 0.006 |  | 0.05(0.01,0.08) | 0.006 |
| MDI | 0.24(-0.26,0.74) | 0.344 |  | 0.38(-0.10,0.87) | 0.124 |  | 0.32(-0.17,0.80) | 0.206 |
| **Age of 6 months** |  |  |  |  |  |  |  |  |
| WAZ | 0.08(0.04,0.11) | <0.001 |  | 0.08(0.05,0.11) | <0.001 |  | 0.08(0.05,0.11) | <0.001 |
| LAZ | 0.04(0.01,0.07) | 0.037 |  | 0.04(0.01,0.07) | 0.024 |  | 0.04(0.01,0.07) | 0.017 |
| WLZ | 0.07(0.04,0.10) | <0.001 |  | 0.07(0.04,0.10) | <0.001 |  | 0.07(0.04,0.11) | 0.006 |
| MDI | -0.07(-0.57,0.43) | 0.784 |  | 0.07(-0.42,0.57) | 0.768 |  | 0.01(-0.49,0.50) | 0.985 |
| **Age of 12 months** | |  |  |  |  |  |  |  |
| WAZ | 0.04(0.01,0.07) | <0.001 |  | 0.05(0.02,0.08) | 0.002 |  | 0.05(0.02,0.08) | 0.001 |
| LAZ | 0.04(0.01,0.07) | 0.038 |  | 0.04(0.01,0.07) | 0.024 |  | 0.04(0.01,0.07) | 0.018 |
| WLZ | 0.03(0.01,0.07) | 0.028 |  | 0.03(0.01,0.07) | 0.028 |  | 0.04(0.01,0.07) | 0.027 |
| MDI | 0.14(-0.38,0.67) | 0.591 |  | 0.29(-0.22,0.80) | 0.270 |  | 0.22(-0.29,0.73) | 0.403 |
| **Age of 18 months** | |  |  |  |  |  |  |  |
| WAZ | 0.05(0.02,0.08) | 0.001 |  | 0.06(0.03,0.09) | <0.001 |  | 0.06(0.03,0.09) | <0.001 |
| LAZ | 0.01(-0.03,0.04) | 0.625 |  | 0.01(-0.02,0.05) | 0.503 |  | 0.01(-0.02,0.05) | 0.435 |
| WLZ | 0.07(0.03,0.10) | <0.001 |  | 0.07(0.03,0.10) | <0.001 |  | 0.07(0.03,0.10) | <0.001 |
| MDI | -0.02(-0.54,0.50) | 0.939 |  | 0.12(-0.39,0.64) | 0.637 |  | 0.05(-0.46,0.57) | 0.835 |
| **Age of 24 months** | |  |  |  |  |  |  |  |
| WAZ | 0.04(0.01,0.07) | 0.014 |  | 0.04(0.01,0.07) | 0.005 |  | 0.04(0.01,0.07) | 0.004 |
| LAZ | 0.01(-0.02,0.04) | 0.517 |  | 0.01(-0.02,0.05) | 0.401 |  | 0.02(-0.02,0.05) | 0.339 |
| WLZ | 0.05(0.02,0.08) | 0.003 |  | 0.05(0.02,0.08) | 0.003 |  | 0.05(0.02,0.08) | 0.003 |
| MDI | 0.15(-0.36,0.65) | 0.565 |  | 0.29(-0.20,0.79) | 0.245 |  | 0.22(-0.27,0.72) | 0.375 |
| **Total** |  |  |  |  |  |  |  |  |
| WAZ | 0.05(0.03,0.08) | <0.001 |  | 0.06(0.03,0.08) | <0.001 |  | 0.06(0.03,0.08) | <0.001 |
| LAZ | 0.02(-0.01,0.05) | 0.080 |  | 0.03(0.01,0.06) | 0.048 |  | 0.03(0.01,0.06) | 0.034 |
| WLZ | 0.05(0.03,0.07) | <0.001 |  | 0.05(0.03,0.07) | <0.001 |  | 0.05(0.03,0.08) | <0.001 |
| MDI | 0.09(-0.24,0.42) | 0.600 |  | 0.23(-0.08,0.55) | 0.147 |  | 0.16(-0.15,0.48) | 0.309 |

Abbreviation: CI, Confidence interval. LAZ, Length for age Z scores; MDI, Mental development index; WAZ, Weight for age Z scores; WLZ, Weight for length Z scores.

^a^ Generalized estimated equation linear model were used to assess the association between maternal weight during pregnancy and physical development in early school aged children.

^b^ Model 1 include the variables of county, sex of infant, maternal age, educational level of parents, household wealth index, occupation of parents.

^c^ Model 2 include the variables of county, sex of infant, gestational weeks at birth, gestational weeks when maternal weight measured, type of prenatal micronutrient supplementation, doses of supplements consumed, maternal age during pregnancy, educational level of parents, household wealth index, occupation of parents.

| **Table S4** Association between gestational weekly weight gain and development of infant (sensitivity analysis, multiple imputation method) ^a^ | | | | | | | | |
| --- | --- | --- | --- | --- | --- | --- | --- | --- |
|  | Weekly gestational weight gain | | | | | | | |
|  | Unadjusted analysis | |  | Model 1 ^b^ | |  | Model 2 ^c^ | |
|  | Coef(95%CI) | *P* |  | Coef(95%CI) | *P* |  | Coef(95%CI) | *P* |
| **Age of 3 months** |  |  |  |  |  |  |  |  |
| WAZ | 0.34(0.06,0.63) | 0.019 |  | 0.32 (0.03,0.60) | 0.028 |  | 0.29(0.01,0.57) | 0.047 |
| LAZ | 0.38(0.08,0.68) | 0.015 |  | 0.39(0.08,0.71) | 0.015 |  | 0.35(0.04,0.67) | 0.030 |
| WLZ | 0.01(-0.29,0.31) | 0.953 |  | 0.01(-0.29,0.30) | 0.961 |  | 0.01(-0.30,0.30) | 0.992 |
| MDI | 13.15(8.74,17.56) | <0.001 |  | 12.03(7.70,16.36) | <0.001 |  | 12.63(8.13,17.12) | <0.001 |
| **Age of 6 months** |  |  |  |  |  |  |  |  |
| WAZ | 0.26(-0.01,0.53) | 0.057 |  | 0.24(-0.03,0.51) | 0.085 |  | 0.21(-0.06,0.48) | 0.133 |
| LAZ | 0.33(0.03,0.64) | 0.033 |  | 0.38(0.08,0.68) | 0.012 |  | 0.34(0.04,0.64) | 0.025 |
| WLZ | -0.03(-0.36,0.30) | 0.873 |  | -0.01(-0.34,0.32) | 0.950 |  | -0.02(-0.35,0.31) | 0.922 |
| MDI | 7.69(3.16,12.22) | 0.001 |  | 6.57(2.19,10.96) | 0.003 |  | 7.64(2.99,12.28) | 0.001 |
| **Age of 12 months** | |  |  |  |  |  |  |  |
| WAZ | 0.43(0.16,0.69) | 0.002 |  | 0.40(0.13,0.66) | 0.003 |  | 0.37(0.10,0.64) | 0.006 |
| LAZ | 0.32(0.01,0.64) | 0.046 |  | 0.33(0.02,0.65) | 0.035 |  | 0.30(-0.02,0.61) | 0.064 |
| WLZ | 0.27(-0.04,0.58) | 0.088 |  | 0.29(-0.02,0.60) | 0.071 |  | 0.28(-0.03,0.59) | 0.079 |
| MDI | 5.26(0.23,10.30) | 0.041 |  | 4.15(-0.83,9.13) | 0.102 |  | 5.01(0.04,9.98) | 0.048 |
| **Age of 18 months** | |  |  |  |  |  |  |  |
| WAZ | 0.18(-0.08,0.45) | 0.168 |  | 0.16(-0.10,0.41) | 0.228 |  | 0.13(-0.13,0.39) | 0.328 |
| LAZ | 0.33(0.03,0.62) | 0.029 |  | 0.34(0.04,0.63) | 0.027 |  | 0.30(-0.01,0.60) | 0.053 |
| WLZ | 0.01(-0.30,0.33) | 0.927 |  | 0.03(-0.28,0.34) | 0.845 |  | 0.03(-0.29,0.34) | 0.876 |
| MDI | 4.00(-0.55,8.56) | 0.085 |  | 2.89(-1.51,7.28) | 0.197 |  | 4.17(-0.45,8.78) | 0.077 |
| **Age of 24 months** | |  |  |  |  |  |  |  |
| WAZ | 0.31(0.05,0.57) | 0.018 |  | 0.28(0.03,0.54) | 0.028 |  | 0.25(0.01,0.51) | 0.050 |
| LAZ | 0.21(-0.10,0.51) | 0.179 |  | 0.22(-0.08,0.52) | 0.149 |  | 0.18(-0.12,0.48) | 0.235 |
| WLZ | 0.16(-0.13,0.46) | 0.271 |  | 0.18(-0.11,0.47) | 0.226 |  | 0.17(-0.12,0.47) | 0.244 |
| MDI | 1.31(-3.16,5.78) | 0.565 |  | 0.19(-4.14,4.53) | 0.930 |  | 1.79(-2.76,6.35) | 0.859 |
| **Total** |  |  |  |  |  |  |  |  |
| WAZ | 0.30(0.08,0.53) | 0.008 |  | 0.28(0.06,0.50) | 0.014 |  | 0.25(0.03,0.47) | 0.029 |
| LAZ | 0.31(0.08,0.55) | 0.010 |  | 0.33(0.09,0.57) | 0.006 |  | 0.29(0.05,0.53) | 0.017 |
| WLZ | 0.08(-0.13,0.29) | 0.444 |  | 0.10(-0.11,0.31) | 0.356 |  | 0.09(-0.12,0.31) | 0.393 |
| MDI | 6.28(3.33,9.24) | <0.001 |  | 5.17(2.39,7.94) | <0.001 |  | 6.25(3.30,9.19) | <0.001 |

Abbreviation: CI, Confidence interval. LAZ, Length for age Z scores; MDI, Mental development index; WAZ, Weight for age Z scores; WLZ, Weight for length Z scores.

^a^ Generalized estimated equation linear model were used to assess the association between maternal weight during pregnancy and physical development in early school aged children.

^b^ Model 1 include the variables of county, sex of infant, maternal age, educational level of parents, household wealth index, occupation of parents.

^c^ Model 2 include the variables of county, sex of infant, gestational weeks at birth, gestational weeks when maternal weight measured, type of prenatal micronutrient supplementation, doses of supplements consumed, maternal age during pregnancy, educational level of parents, household wealth index, occupation of parents.
